# A High‐Resolution Microscopy System for Biological Studies of Cold‐Adapted Species Under Physiological Conditions

**DOI:** 10.1002/smtd.202401682

**Published:** 2024-12-15

**Authors:** Anne‐Pia M. Marty, Edward N. Ward, Jacob R. Lamb, Francesca W. van Tartwijk, Lloyd S. Peck, Melody S. Clark, Clemens F. Kaminski

**Affiliations:** ^1^ Department of Chemical Engineering and Biotechnology University of Cambridge Phillipa Fawcett Drive Cambridge CB3 0AS UK; ^2^ British Antarctic Survey High Cross, Madingley Road Cambridge CB3 0ET UK

**Keywords:** cold‐adaptation, extremophiles, optical microscopy, super‐resolution, temperature

## Abstract

The Antarctic seabed harbors significant biodiversity, and almost 90% of oceanic environments are permanently below 5 °C (i.e., deep sea and polar regions). However, organisms whose entire lifecycle occurs around 0 °C are understudied, leaving this large and diverse proportion of the global biome poorly understood. To address this question at the cellular level, tools are required for high‐resolution imaging of biological systems under physiological conditions. This poses severe technical challenges. High‐resolution imaging objectives require short working distances and immersion media, causing rapid heat transfer from the microscope to the sample. This affects the viability of live specimens and the interpretability of results. Here, we present a method for high‐fidelity imaging of live biological samples at temperatures of around, or below, 0 °C. It relies on hardware additions to traditional microscopy, namely as a cooling collar, 10% ethanol as an immersion medium, and nitrogen flow to reduce condensation It can be straightforwardly implemented on different microscopy modalities, including super‐resolution imaging. The method is demonstrated in live cell cultures derived from Antarctic fish and highlights the need to maintain physiological conditions for these fragile samples. Future applications include evolutionary biology, biophysics and biotechnology.

## Graphical abstract

Regions of the world permanently below 5 °C harbor significant biodiversity and understudied molecular mechanisms. This knowledge gap originates from the requirement for high‐resolution microscopy methods that maintain cold sample conditions while achieving subcellular resolution. Here, a technical solution is presented, evaluate its thermal and optical performance, and apply it to imaging Antarctic fish samples.

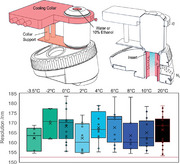

## 1 Introduction

The fundamental processes of life are governed by elementary chemical and physical mechanisms, such as diffusion, transport, and chemical reactions, all of which depend sensitively on temperature. At different scales, from molecular to cellular levels, temperature also impacts density, viscosity, gas solubility, etc. Since these physical properties are interdependent, and all are governed by temperature, most life forms have evolved to operate over their respective optimal temperature ranges, outside of which they cannot survive.^[^ ^1^ ^]^

While biological processes at normothermic conditions (e.g., 37 °C in human physiology) are well‐characterized, the quality of model predictions are often less good for organisms with lower normothermic temperatures. This decline in quality is particularly evident at the Arrhenius break temperature (ABT), which typically ranges between 2 and 5 °C, depending on the specific biological process,^[^ ^2^, ^3^ ^]^ below the ABT, the relationship between the logarithm of biochemical reaction rates and the inverse of temperature deviates from linearity, indicating a fundamental change in the rate‐limiting steps driving these reactions. This deviation is evident also from the disproportionately increasing timescales below the ABT for life‐sustaining processes like protein folding, cell division, and animal development.^[^ ^2^ ^]^

Research into the biophysical adaptations of life at temperatures around 0 °C is not merely of fundamental academic interest but is of ecological relevance for understudied environmental conditions. Notably, over 88% of the oceanic volume lies at depths exceeding a thousand meters, beneath which the temperatures hover around 4 °C.^[^ ^4^ ^]^ Contrary to cold habitats on land, where direct exposure to the sun can make temperatures rise by tens of degrees, the thermal inertia of water makes marine temperature changes minimal. The Southern Ocean's surface temperatures range between −2 and 2 °C all year round.^[^ ^3^ ^]^ The vast and diverse biome of cold oceans is home to numerous species whose survival strategies and basic life mechanisms remain poorly understood, due to the inadequacy of existing models describing life at such low temperatures. Much of this biome is at risk from global warming and rising water temperatures, and efforts to improve understanding of cold adaptation are therefore both timely and important.

Research into cold‐adapted biological systems has thus far been limited by the fact that traditional biotechnological tools, including various forms of microscopy, are not designed to function well at low temperature. In the case of microscopy, image quality suffers, and it is difficult to maintain cold‐adapted samples at their physiological temperature. In this work, we address these challenges, developing methodologies and tools capable of accurately probing biological phenomena at the subcellular level at near‐freezing conditions.

**Figure 1:**
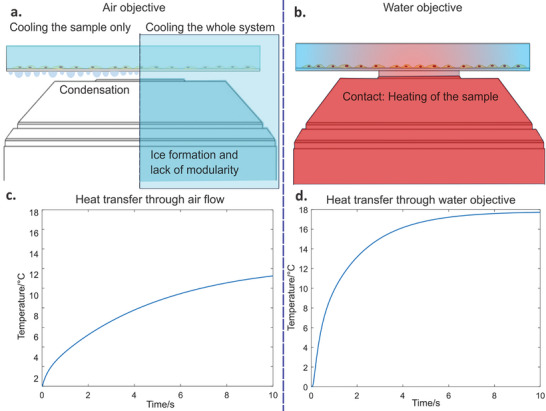
Heat transfer poses challenges during imaging of samples at temperatures significantly below ambient. a) Left: Using an air objective while keeping the sample cold but the microscope at ambient temperature leads to condensation on optical surfaces. Right: Condensation is prevented by operating the microscope in a cooled environment; however, the risk of ice formation severely limits operational freedom. b) Using a liquid‐immersion objective maintained at ambient temperatures heats the sample through thermal contact. c,d) Simulations show that due to the very short working distance of high‐resolution objective lenses, heat transfer through either convection—with an air immersion lens—or conduction—through a liquid immersion lens—rapidly heats the sample at the focal plane of the imaging lens. In both simulations, the sample chamber is cooled externally, but the imaging lens remains at ambient temperatures. More details on simulation parameters are given in Figure S1 (Supporting Information).

Achieving cooling at the focal plane of a microscope presents several issues, such as ice formation, condensation, and sample heating (**Figure** **1**). One could house an entire microscope system in a cold room, but this restricts the use of expensive equipment to one type of application only. It also makes changing temperature conditions difficult and slow, as thermal inertia means hours of settling time are required. Condensation problems can also occur during warming up or cooling down of microscope components as they transition through atmospheric dew points. This is especially critical during maintenance or servicing of cold laboratories, when the temperature is brought to ambient levels. Finally, using samples in aqueous solutions or simply the presence of experimenters near the apparatus introduce humidity which will freeze over any exposed surface.

Fully enclosed sample chambers from commercial suppliers (Linkam, MicrOptic, Instec) were used in previous work to reach low temperatures and prevent condensation, but this has critical disadvantages, relying on low resolution widefield microscopy with long working distance objectives,^[^ ^4^, ^5^, ^6^ ^]^ or not permitting sample cooling and high resolution imaging to be performed at the same time.^[^ ^7^, ^8^ ^]^ In other reported work, cooling was achieved through use of a specially designed sample chamber containing a Peltier element, a water circulation system, and a sample holding surface with high thermal conductivity.^[^ ^9^, ^10^ ^]^ These chambers introduce additional glass surfaces between the sample and the imaging lens, increasing working distance and again requiring the use of low numerical aperture (NA) lenses. Furthermore, optical mismatches introduce spherical aberrations and lower the image quality. In a few reports, use of immersion media and high NA lenses were presented, but the temperatures reached were limited because of the risk of freezing.^[^ ^10^ ^]^ Table S1 (Supporting Information) offers a summary of previous work on the topic of imaging samples contained in the cold. No single solution achieves subzero temperature and a high enough resolution to resolve subcellular detail during microscopic imaging, while keeping samples alive.

Using a cooling stage rather than an enclosed chamber, on the other hand, introduces an air gap between the imaging lens and the cooled sample and this inevitably generates condensation (Figure 1a). It is not easily possible to mitigate this problem. For example, attempting to prevent condensation by blowing a stream of dry gas into the space between the sample and the objective results in the sample quickly reaching the air temperature via convective heating. A simulation of this effect is shown in Figure 1c. The material properties and dimensions used in the simulations shown in Figure 1c,d are representative of commonly used equipment. While variations may occur between experiments, these parameters remain broadly applicable. Further details on the simulation method are provided in Figure S1 and Table S2 (Supporting Information). The use of an immersion medium between the sample and imaging lens circumvents the condensation problem associated with cooling stages, but massively increases heat conduction from the objective to the sample. This leads to the sample almost immediately reaching the temperature of the lens as shown in the simulation presented in Figure 1d. Therefore, existing solutions such as cooling stages can be useful to keep the sample cold but cannot simultaneously achieve conditions for high‐quality imaging and the maintenance of low temperatures within the field of view.

To address this, we propose a design for a cooling chamber (Figure S2, Supporting Information) and propose modifications to standard optical microscopes that permit the imaging of samples while maintaining them at their physiological temperature around 0 °C. The methods presented here prevent issues of condensation and heat conduction and maintain image resolution while keeping fragile live samples viable.

## 2 Experimental Section

### 2.1 Thermal Simulations

Heat transfer simulations were performed using the MATLAB PDE toolbox, generating a model of the objective to sample contact with the dimensions and physical values as stated in Figure S1 and Table S2 (Supporting Information), respectively. The model was run for 30 000 steps, with 1000 steps s^−1^.

Simulations for the thermal performance of the collar are shown in Figure S3 (Supporting Information). Calculations were performed using the Fusion360 thermal simulation workspace. All.obj files, code scripts, and simulation parameters are available on github.com/piamrt/coolmicroscope. Thermal loads were set to 20 °C air temperature, and −15 °C collar temperature.

### 2.2 Refractive Index Measurements

Refractive indices were measured using an Abbe ‘60′ Refractometer from Bellingham & Stanley Limited. The refractometer was coupled to a Cole Palmer Polystat 12104‐05 water chiller and pump system, circulating a 70:30 water‐glycerol mix. The light source was a 4‐wavelength LED source (LED4D245 Thorlabs) with a DC4104 LED driver.

### 2.3 Hardware Design

The collar was manufactured from custom specifications by the company Instec. The collar holder and the air inlet were designed using the Fusion360 software. The resulting Obj output files were sliced using the Ultimaker Cura software and printed in PLA (Ultimaker) on Ultimaker printers. All design files are accessible on github.com/piamrt/coolmicroscope.

The sample chamber shown in Figure S2 (Supporting Information) was also designed with Fusion 360 and machining steps were defined in the manufacturing mode of the software. The chamber was machined from aluminum with a Siemens XYZ Machinetools XYZ 500LR CNC mill. The chamber was designed to accommodate Lab Tech 8‐ and 4‐well chambers, as well as ibidi µ‐slides and 35 mm round culture dishes.

### 2.4 Temperature and Power Measurements

8‐well 1.7 mm glass‐bottom cell‐culture well‐plates (Lab‐Teck) were filled with 500 µL water to simulate culture medium. In‐well temperatures were recorded using a TC‐08 thermocouple data logger (Piotech). The probes were type K exposed junction thermocouples with a tip diameter of 1.5 mm. Small holes were pierced into the lid of the culture chambers. Probes were attached to the wells to be in contact with the bottom of the well and the microscopes were focused so that the tip of the probe was in focus. Data were acquired in continuous data acquisition mode at a sampling rate of one datapoint per second. The power of the laser was measured with a Thorlabs PM100D Power Meter and an S130C probe (Thorlabs). Results presented correspond to the average of 5 repeat measurements.

### 2.5 Image Acquisition and Processing of Beads on a Widefield Microscope

For the acquisition of bead images, the microscope used was as described in Young et al. 2016,^[^ ^11^ ^]^ and operated in widefield mode. Monolayers of 0.1 µm diameter TetraSpeck beads (ThermoFisher) were produced using a 10^−4^ dilution of the stock solution. Fluorescence image stacks of the beads were acquired using a widefield microscope, assembled recorded over the field of view, with a step size of 0.02 µm along the optical axis. The code for generating image stacks, segmenting the beads, and calculation of their full width at half maximum (FWHM) is available on github.com/piamrt/coolmicroscope.

### 2.6 Super‐Resolution Imaging

Structured illumination microscopy (SIM) was the system used for super‐resolution imaging. The microscope was built according to the instructions in Young et al. 2016.^[^ ^11^ ^]^ SIM is a method relying on the generation of Moiré Patterns from the interference between the sample spatial frequencies and the patterning patterns. This method typically doubles the resolution of diffraction‐limited microscopy. The SIM system uses a IX71 microscope stage (Olympus) and three laser sources for excitation: 488 nm (iBEAM‐SMART‐488, Toptica), 561 nm (OBIS 561, Coherent), and 640 nm (MLD 640, Cobolt). Patterning was done with a ferroelectric binary Spatial Light Modulator (SLM) (SXGA‐3DM, Forth Dimension Displays) and polarization was controlled with a Pockels cell (M350‐80‐01, Conoptics). The camera used was an sCMOS camera (C11440, Hamamatsu), the raw image acquisition and recording was managed by the HCImage software (Hamamatsu) and the hardware was synchronized using a custom LabView program (available upon request).

### 2.7 Fixed Samples

Fixed cells were used to compare the quality of imaging across a temperature range, on a biological sample that would not dynamically change with temperature. Samples of mammalian (Vero) cells were fixed using 4% Paraformaldehyde (PFA) and 0.1% glutaraldehyde in PBS for 10 min at room temperature. Cells were permeabilized using Triton x‐100 0.5% in PBS also for 10 min at room temperature. The subsequent blocking step used 10% goat serum in PBS for 30 min at room temperature. The Primary antibody was a Mouse AB13120 β‐tubulin‐targeting antibody and the secondary antibody was Rabbit AB6046 (Abcam). Both were diluted 1:400 in PBS with 2% Bovine Serum Albumin (ThermoFisher) and 0.005% Triton, and each was incubated 1 h at room temperature, the last step being performed in the dark.

### 2.8 Antarctic Fish Cell Culture and Live Imaging

Primary cell cultures were obtained from dissected tissues of *Harpagifer antarcticus* (unpublished methods). All *H. antarcticus* used in the experimental work were collected at Rothera Research Station, Adelaide Island, Antarctic Peninsula (67°34′07″ S, 68°07′ 30″ W) by SCUBA divers during the austral summer. The fish were returned to the UK and maintained in a recirculating aquarium at temperatures close to 0 °C until required. Fish were collected under a permit (BAS‐S7‐2022/01) granted under Section 7 of the Antarctic Act 1994. Before dissections were performed for cell cultures, all fish were killed according to Home Office UK schedule 1 requirements. Cell monolayers were stained with MitoTracker green according to the manufacturer recommendations. Samples were imaged at 2 °C, brought to 20 °C for 1 h and then imaged again at 2 °C using a SIM.^[^ ^12^ ^]^

Acquired images were reconstructed using the Fiji LAG‐SIM package, available from the Fiji Update sites. LAG SIM runs on the fairSIM^[^ ^13^ ^]^ package and provides an interface allowing iteration through different parameters and processing of batches of data.

The contrast of the reconstructed images was enhanced manually, using the Fiji image processing tools “Enhance Local Contrast” and “adjust Brightness/Contrast” and the images were exported in JPEG format to be analyzed in CellProfiler.^[^ ^14^ ^]^ The following CellProfiler pipeline was used to analyze the mitochondrial phenotype: ColorToGray > RescaleIntensity > MedianFilter >IdentifyPrimaryObjects > MeasureObjectSizeShape > ExportToSpreadsheet > OverlayObjects > SaveImages.

The resulting CSV files were then imported into MATLAB for plotting and to perform Student's *t*‐tests.

### 2.9 Statistical Analysis

**Figure 2:**
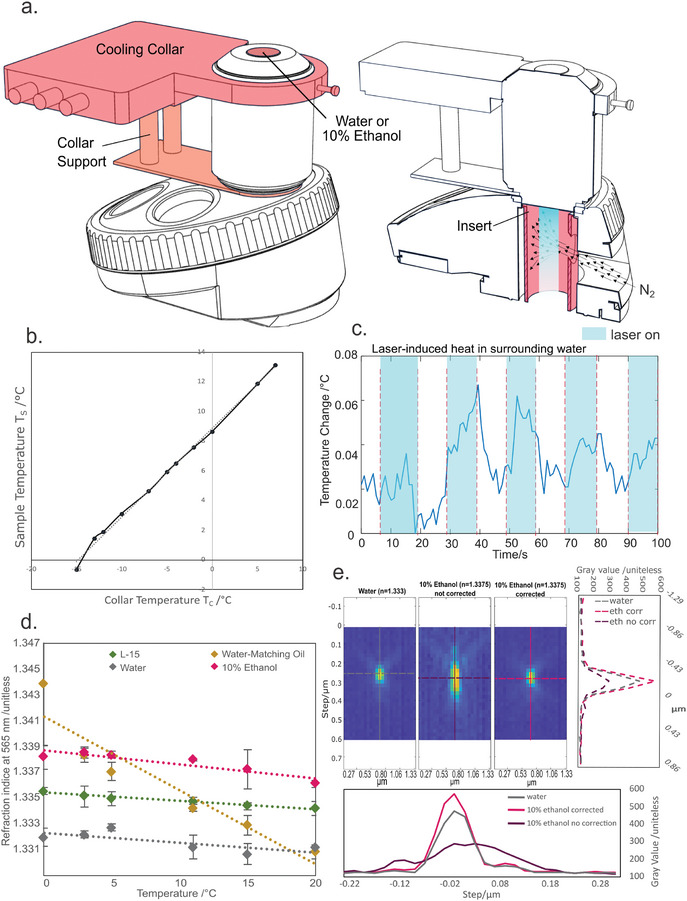
The use of a cooled water‐immersion lens allows for imaging at subzero temperatures with no loss in image resolution. a) Left: A supported cooling collar attached to the top of the objective keeps the image plane at the desired temperature while the microscope remains at room temperature. Right: To prevent condensation of water at the rear of the objective, the air behind the objective lens is purged with dry gas. b) Temperature reached at the focal volume plotted as a function of the collar temperature. Straight line fit: *T*_S_ = 0.59T_C_ + 8.87. c) Heat generated by the laser excitation resulted in a temperature change of <0.1 °C. d) Temperature dependent variation in refractive index for objective immersion liquids. Optimal 3D imaging of samples requires an objective immersion liquid with a refractive index equal to that of the sample (L‐15 data points). 10% Ethanol/water mixture shows minimal variation in refractive index across the range of temperatures tested while maintaining transparency without freezing below 0 °C (*N* = 3). e) *x*–*z* projections of point spread functions and corresponding center line profiles in *x* and *z* measured with water, and 10% ethanol/water mixtures as immersion media. The spherical aberrations induced by refractive index mismatches using the ethanol/water mixture (middle panel) can be corrected for using the correction collar on the water immersion lens. Representative data from *n* = 40 repeats.

**Figure 3:**
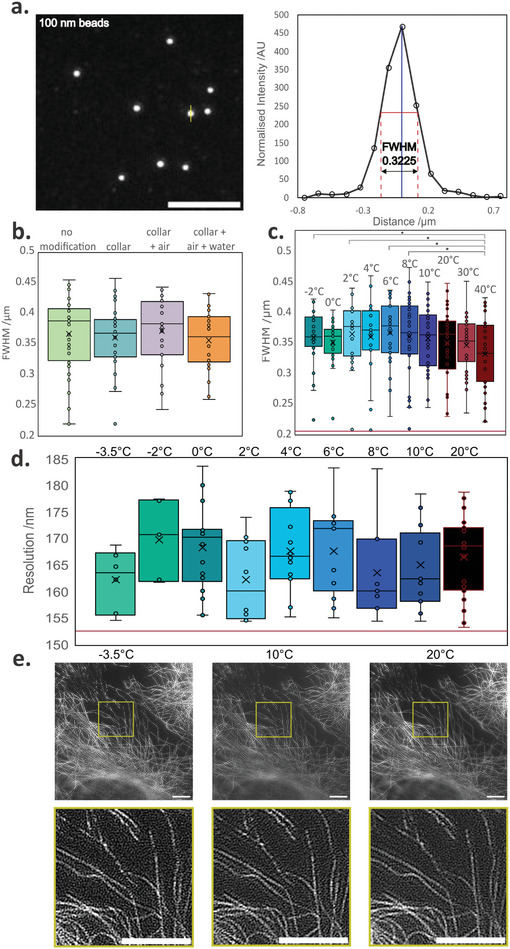
The system performs with near diffraction limited resolution across a 20° temperature range. a) Diffraction‐limited image of bead monolayer (left) and full‐width at half maximum plot of indicated bead (right). No significant differences in PSF size were observed under different system configurations (one‐tailed unpaired Student's *t*‐test, *N* = 30). c) PSF size remains consistent across a 40 to −2 °C temperature range (one‐tailed unpaired Student's *t*‐test, *N* = 26). The red line indicates the diffraction limit at 214 nm. d) Image resolution estimates from single‐image Fourier ring correlation (FRC). Estimated resolution stays comparable across the whole temperature range (*N* = 7). The red line indicates the diffraction limit at 153 nm. e) Reconstruction of SIM images from fixed microtubules at 20, 10, and −3.5 °C. Scale bar is 5 µm for all images. The sign* indicates a *p* value of <0.05, all the other relationships are nonsignificant.

The temperature curve and impact of the laser presented in **Figure** **2**b,c are subsets of 3 repeats that required no preprocessing. The fit for the temperature curve was done using Microsoft excel. The refraction indices measurement presented are averages and standard deviations from 3 repeats. The point spread function profiles are representative data from three acquisitions of 20–30 beads in the field of view. The selection of the beads for analysis was done using the Fiji function “Find Maxima” and setting the prominence manually for each image, leading to the extraction of a minimum of 40 bead images per condition. The same bead identification method was applied to the beads used for **Figure** **3**b,c but the minimum number of beads extracted was *n* = 30 for Figure 3b and *n* = 26 for Figure 3c. The Fourier ring correlation images come from 3 repeats acquisitions. The images were reconstructed using the LAG‐SIM plugin in Fiji and then analyzed using Koho et al's^[^ ^15^ ^]^ published code in python. All the output values were exported to Microsoft Excel. The Single image FRC code can generate NaN outputs which made the individual sample size for each condition vary but each condition had at least 7 data points and at most 24. The biological application images were processed as described in Section 2.8, and are composed of 19 cells over 3 repeat experiments. All the statistical tests are one‐tailed, unpaired Student's *t*‐test.

### 2.10 Ethical Statement

The Fish used for this study were collected under permit (BAS‐S7‐2022/01) granted under Section 7 of the Antarctic Act 1994. For the purpose of open access, the authors have applied a Creative Commons Attribution (CC BY) licence to any Author Accepted Manuscript version arising from this submission.

## 3 Results and Discussion

Our design was informed by model predictions of a high rate of heat transfer between the objective lens and the sample through the immersion medium (water or 10% ethanol; Figure 1d). We decided to use this phenomenon for maintaining the correct temperature of the sample around the focal volume. A cooling element was attached close to the tip of the objective (Figure 2a). Its purpose was to cool the objective to a temperature of around −15° C and for the objective to act as a heat sink with high thermal mass. The cooling of the collar was achieved through a thermoelectric (Peltier) cooling system arranged around the objective (Figure 2a) containing brass ring insert that was in tight thermal contact with the objective. The thermoelectric elements permit cooling down to −30 °C. A water circulation system was used to remove heat from the system. We did not notice any effect of water circulation in image quality, e.g., through flow induced vibrations. This is investigated further in Section 4. Subsequent heat transfer from the sample to the objective reduced the sample temperature to the desired range of around 0 °C (Figure 2b). Thermal simulations show that a cooling the collar must theoretically be maintained at −12.6 °C to allow the temperature of the sample to reach 0 °C at the point of focus (full details are given in Figure S3 and corresponding text, Supporting Information). Variations in objective shapes and size mean that the use of different objective models requires new calibration curves to be established. Figure 2b shows data for a 60x water objective (Olympus UPLSAPO60XW, used for all experiments presented here). TC‐08 thermocouple probes (PicoTech) were used in an 8‐well glass‐bottom (0.17 mm tick) dish to record the temperature near the focal point of the objective, verifying that sub‐0 °C temperatures are reachable in the sample volume.

To prevent condensation at the back of the objective, dry nitrogen was injected into the turret using a 3D‐printed insert and a gas line (Figure 2a). To prevent the extra weight of the collar from straining the objective, we designed a 3D‐printed support structure to stabilize the objective (Figure 2a). Further details can be found in Figure S4 (Supporting Information).

A remaining concern for us was the potential heating effect of the sample through heat transfer from the illumination light. We measured the temperature rise in water adjacent to the focal spot of a 488 nm laser set to 0.45 ± 0.01 mW, carefully avoiding direct heating of the temperature probe with the excitation light. The power of the laser reaching the sample was not determined but the output power was chosen to be relevant for most biological applications. The induced temperature change reached a maximum of 0.067 °C (Figure 2c). The difference in temperature when the laser was on or off was too small to warrant further efforts to mitigate effects of laser induced heating of the sample.

Finally, the correct choice of immersion medium is critical to obtain optimal resolution and light collection efficiency. Traditional immersion media, such as silicon‐ and mineral‐oils, become viscous when cooled down, hindering movement of the sample over the objective. Below 0 °C water is unsuitable for imaging because of freezing. Adding salts to the water would only lower the freezing point by a couple of degrees and is likely to erode the protective coating of the objective. A suitable immersion medium for this system needs to combine the qualities of having a freezing point below 0 °C and a refractive index matching that of water over the range of temperatures the system is operated over. Furthermore, the liquid needs to be chosen to avoid damaging the coatings of the objective. Commercially available solutions of water‐matching oil (Cargille) were compared to water, culture media, and water‐based dilutions of ethanol. A 10% dilution of ethanol in water was found to be the best candidate, yielding a refractive index of 1.34 at 565 nm and 0 °C (Figure 2d), with a freezing point of −4 °C. The refractive index of this mixture was found to stay nearly constant over the visible wavelength range (Figure S5a,b,c, Supporting Information). The refractive index of 1.34 is 0.0045 away from the optimum for the lens (namely *n* = 1.333 for water). This does change the PSF shape and introduces aberrations, but these can be fully corrected for by changing the correction collar to +0.015 mm of glass, making 10% ethanol a suitable immersion medium (Figure 2e). Furthermore, the mismatch between the refractive index of the cell culture medium (green line in Figure 2d) and the ethanol/water mixture (magenta line in Figure 2d) is never greater at any temperature, than that between water as an immersion medium (gray line in Figure 2d) and the cell culture medium. This means that we are close to design conditions with our proposed immersion medium for cold imaging.

### 3.1 Performance of the System

We next assessed whether microscope resolution was affected by mechanical distortion or movement of components caused by our modifications. We compared the performance of the original system without modifications, and that of the instrument with successive additions of the collar, the nitrogen flow, and circulation of the water in the collar. The resolution was assessed by measuring the full width at half maximum (FWHM) of cross sections of 0.1 µm TetraSpeck bead images obtained at 488 nm excitation wavelength using a 60x, NA = 1.2 water objective (Figure 3a). A Student's *t*‐test (one‐tailed, unpaired) was performed, and the results are shown in Figure 3b. No statistically significant differences in resolution were seen to occur (*p* < 0.25 for all cases).

We then investigated whether each modification introduced vibrations during imaging at high frame rates. Stacks of images of 170 nm Tetraspeck beads were acquired 60 Hz frame rates and collected for each condition. From line profiles of the recorded point spread functions and fitting, we verified that the PSF peak stayed within one pixel (Figure S6a,b, Supporting Information). We performed a fast Fourier transform (FFT) analysis of the peak displacements from the origin to determine the frequency and magnitude of vibrations introduced by each modification (Figure S6c, Supporting Information). Mostly, amplitudes of vibrations were contained within one standard deviation of those recorded for control conditions (Figure S6d, Supporting Information). Additionally, no peaks were observed in the frequency spectra, indicating no fixed vibration was present in the system.

As the temperature of the objective decreases, we expect a slight thermal contraction to occur in the objective. Objective barrels used were made of brass.^[^ ^16^ ^]^ This has a linear coefficient of thermal expansion (α) of ≈19 × 10^−6^ K^−1^. Cooling the objective to −20 °C could thus generate a contraction of up to 30 µm over the full length of the barrel. Because the cooling happens symmetrically around the objective, the potential aberrations would only affect the radially symmetric Zernike polynomials, i.e., defocus and spherical aberrations. These can be corrected for by the correction collar of the objective. Furthermore, expansions/contractions of this order are not unusual in routine microscope practice: Objectives used for the imaging of live mammalian cell lines are routinely heated to keep the sample temperate. For example, commercial Okolab objective heaters raise temperatures by up to 17 °C above ambient, introducing thermal expansion of up to 20 µm. Ensuing aberrations are routinely corrected for using the objective correction collar.

A phenomenon to consider when imaging at different temperatures are potential changes to fluorophore photostability. This topic has been investigated in detail for fluorophores at cryogenic temperatures. Both fusion proteins and synthetic fluorophores tend to exhibit increased stability at low temperature.^[^ ^17^, ^18^, ^19^, ^20^ ^]^ This is associated with a decrease of rotational degrees of freedom available to the molecule, facilitating electron delocalization and hence fluorophore brightness. Collisional quenching is reduced and other nonradiative processes suppressed.^[^ ^21^ ^]^ We expect similar (although less pronounced) effects at 0 °C.

Overall, however, we observed no significant change in optical performance compared to control conditions (Figure 3). The FWHM of obtained point spread functions were recorded at temperatures ranging from 40 to −2 °C using 0.1 µm TetraSpeck beads at an excitation wavelength of 488 nm and using a 60x water objective. The averages of several PSFs were plotted (Figure 3c), and the corresponding averages for the FWHM were compared for each temperature. Temperatures between −2 and 40 °C were compared. Two temperature points above room temperature (RT) were included to compare the performance of cold microscopy to that of heating the objective and the sample. While heating the objective and sample to 37 °C is a common practice in long‐term imaging of live mammalian cells,^[^ ^22^, ^23^ ^]^ the impacts on optical properties of heating the objective 17 °C above room temperature is rarely considered. The differences in FWHM were not significant for the PSFs obtained at low temperatures, as *p*‐values for over the entire range tested were above 0.113 when compared to the RT control (one tailed, unpaired Student's *t*‐test). Cross‐comparison of all the conditions highlighted the only significant difference between conditions to be between the 40 °C and the cold temperatures (−2, 2, 6, and 8 °C). Across the cold temperatures, there were no statistical differences (Table S3, Supporting Information).

To demonstrate the suitability of the method for super‐resolution imaging, and to further test the impact on resolution, Fourier ring correlation (FRC),^[^ ^24^, ^25^ ^]^ was used to assess the resolution of structured illumination microscopy (SIM) data obtained at low temperature. We used fixed samples for this experiment. The resolution was calculated using the single‐image FRC method developed by Koho et al.^[^ ^15^ ^]^ and setting the threshold criteria to “half‐bit.” Rather than using two identical images, this approach divides a single original image into four subimages, applying a Hamming window to each to minimize artificial correlations and edge effects. Images were taken of microtubules at −3.5, ‐2, 0, 2, 4, 6, 8, 10 °C, and at room temperature (Figure 3). The order of recorded temperatures was randomized and changed across repeats to reduce the chances of bleaching impacting the measurements. The Fourier resolution change was insignificant across the temperature range, with *p*‐values for the low temperature data above 0.134 in comparison to the room temperature result (one tailed, unpaired Student's *t*‐test). One reason for the apparent stability of imaging performance could be that the worsening effect of optical aberrations is offset by improvements in the increasing signal to noise ratio when fluorophores are imaged at lower temperature. The latter appears supported by the greater statistical difference observed in the higher temperature result compared to the low temperature case (Table S3, Supporting Information).

### 3.2 Long‐Term Operation

To achieve the best cooling performance and avoid strain on the Peltier system, it is advisable to maintain the circulating water in sufficient quantity and keep the reservoir filled with at least 1 L of circulating liquid. To reach temperatures below 0 °C, ice can be added to the circulating water to achieve optimal cooling of the Peltier element. The collar adds extra weight to the turret, making it necessary to tighten the height adjustment mechanism. A comparison of optical performance over a 6‐month period of regular system use (Figure S7, Supporting Information) reveals no significant changes in image quality.

## 4 Application

Cold‐adapted organisms are one example of biological samples for which low‐temperature live imaging is a necessity. Antarctic marine fauna has adapted to a very stable and narrow thermal environment. Thermal tolerance studies on such psychrophilic species reveal a 50% failure in essential biological functions at temperatures in the 2–3 °C range.^[^ ^2^, ^26^, ^27^, ^28^ ^]^ To illustrate the need for a cold imaging system, we have chosen to visualize cells from the Antarctic plunder fish *Harpagifer antarcticus*, which is endemic to the Southern Ocean.^[^ ^29^ ^]^ The entire lifecycle of *H. antarcticus* occurs along the Antarctic Peninsula. Temperatures in this environment typically range only between −1.9 and 2 °C. Although it is possible to acclimate this species, at least short‐term, to 3 °C,^[^ ^30^ ^]^ this fish, along with other Notothenioids in the Southern Ocean, has an upper lethal thermal threshold of 5–6 °C.^[^ ^31^, ^32^ ^]^ We have developed cell culture protocols for *H. antarcticus* but were not previously able to study the cells under physiological conditions due to the lack of suitable optical equipment. Therefore, the cellular mechanisms of adaptation to the cold and consequential molecular and organelle responses to warming in this species, or any polar marine organism, are largely unknown.

**Figure 4:**
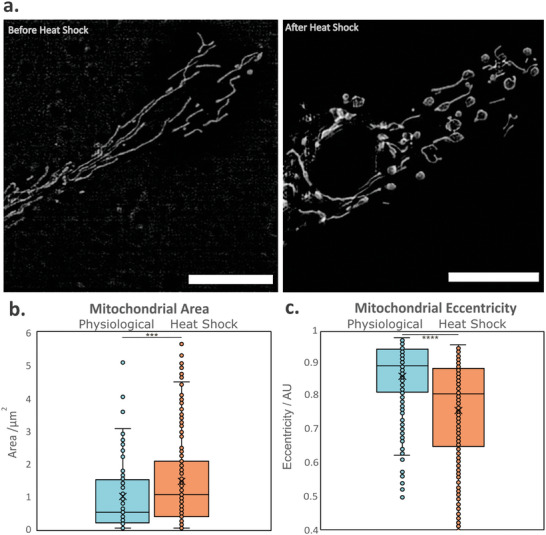
Cold adapted species need to be imaged at their physiological temperature to prevent heat shock. a) Images of mitochondria from cold‐adapted cells (primary cell culture of *Harpagifer antarcticus* gonad) acquired at 2 °C before (left) and after (right) exposure to room temperature for 1 h. Scale bars are 10 µm in both images. b) Area and c) eccentricity of the mitochondria in physiological condition pre‐ and post temporary exposure to room temperature. *n* = 19, *N* = 3. A significant change in mitochondrial morphology associated with heat shock is observed even after cells have returned to physiological temperatures. This indicates that even short imaging sessions on a noncooled microscope would perturb natural homeostasis and prevent meaningful in vivo study. Three stars indicate a *p* value <0.001 (one tailed, unpaired Student's *t*‐test).

To demonstrate that our method improves sample viability during imaging, we compared the impacts of warming in these thermally sensitive samples by imaging at 20 °C on a conventional system and at 2 °C using the methods presented here. Primary cell cultures (gonad) from *H. antarcticus* were grown at 2 °C for 20 days. The cells were then stained using MitoTracker Green and imaged at 2 °C, as a control observation of cold‐adapted cells in physiological conditions. The cells were then left at 20 °C for 1 h, approximately the duration of a simple in vivo imaging session. Samples were then cooled back to 2 °C and imaged again. Mitochondria became more rounded and fragmented as a result, highlighting the damage induced by a room‐temperature microscopy experiment on these sensitive samples (**Figure** **4**). We noticed especially that the stress response manifests as a change in the balance between mitochondrial fission and fusion, which is a well‐described phenotype of mitochondrial stress and apoptosis.^[^ ^33^, ^34^ ^]^ Two metrics best describe this change of phenotype: mitochondrial eccentricity and area. Eccentricity is the measure of how round an object is with the parameter ranging from 0, a perfect sphere (no eccentricity), to 1, characterizing a line (fully eccentric). Healthy mitochondria typically display a string‐like shape.^[^ ^33^ ^]^ When the damaged mitochondria become more circular and fragmented, the change in area and eccentricity becomes significant (Figure 4). Student's *t‐*test between the two conditions show *P*_area_ = 0.001 and *P*_eccentricity_ = 2.523e^−19^.

The cells were left to recover for 24 h at 2 °C and were imaged the next day, but none survived. This further illustrates how acute heat shock, even when temporary, can irreversibly damage cold‐sensitive samples.

## 5 Conclusion

This work proposes a design for a cooling module that enables high‐resolution and super‐resolution microscopy of samples maintained at, or near, 0 °C, and permits observations to be made in live cells. The hardware modifications required to adapt a traditional imaging system for this purpose are relatively minor and our design is compatible with a range of microscope system. The method can thus readily be adopted by other researchers interested in studying biological systems adapted to the cold. We have demonstrated the application of our method for live and super‐resolution imaging live cells derived from the Antarctic fish *H. antarcticus*. This work further exploration of additional biophysical properties and diverse psychrophilic species. Beyond polar biological sciences, this novel method enables real‐time observation of proteostasis mechanisms, including protein folding, condensation, activity, and denaturation. It also offers potential applications in studying biophysical processes such as liquid–liquid phase transitions or cytoskeletal protein polymerization under varying temperatures. Furthermore, the method holds promise in biotechnology and the food industry, with examples including the cold storage stability of cells, tissues, and protein‐based drugs. Examples include cold storage stability of cells, tissues or protein‐based drugs, and transport of transplanted organs, or countless other questions where a microscopic understanding of the effects of cooling and temperature cycling is of interest.

## Conflict of Interest

The authors declare no conflict of interest.

## Author Contributions

A.‐P.M.M.: Methodology, Investigation, Formal analysis, Visualization, Writing – Original Draft. E.N.W.: Conceptualization, Software, Writing – Review and Editing. J.R.L.: Software. F.W.v.T.: Writing – Review & Editing. L.S.P.: Supervision, Writing – Review and Editing. M.S.C.: Project conception, Supervision, Funding acquisition, Project administration, Writing – Review & Editing. C.F.K.: Project conception, Supervision, Funding acquisition, Project administration, Writing – Review & Editing. All authors read and approved the final version.

## Supporting information



Supporting Information

## Data Availability

The data that support the findings of this study are openly available in github at https://www.github.com/piamrt/coolmicroscope, reference number 0.
