# Supplementary material for: A High‐Resolution Microscopy System for Biological Studies of Cold‐Adapted Species Under Physiological Conditions
Source: Small Methods. 2024 Dec 15;9(5):2401682. doi: 10.1002/smtd.202401682 (PMC12103227; doi:10.1002/smtd.202401682)

1. **Tables of reagents, software and equipment**

**1.1 Reagents**

| **Chemical** | **supplier** | **Catalogue number** |
| --- | --- | --- |
| MitoTracker green | Invitrogen | M7512 |
| 16% Paraformadlehyde | Thermo Scientific | 28906 |
| Triton | Thermo Scientific | 28314 |
| Glutaraldehyde | Sigma Aldrich | G7776 |
| Mouse AB13120 β-tubulin | Abcam | AB13120 |
| Rabbit AB6046 Antibody | Abcam | AB6046 |
| 0.1µm TetraSpec | Invitrogen | T7279 |
| Water-matching oil | Cargille | 3421 |

**1.2 Software**

| **Software** | **company** | **link** |
| --- | --- | --- |
| MATLAB R2023a | MathWorks | https://uk.mathworks.com/products/matlab.html |
| Fusion360 | Autodesk | https://www.autodesk.com/products/fusion-360 |
| Fiji | Fiji | https://imagej.net/Fiji |
| CellProfiler 4.2.6 | CellProfiler | https://cellprofiler.org/ |
| Ultimaker Cura | Ultimaker | https://ultimaker.com/software/ultimaker-cura/ |
| Picolog6 | picotech | https://www.picotech.com/data-logger/tc-08/usb-tc-08-logger-picolog-6-software |
| HCImage | Hahahatsu | https://hcimage.com/ |
| LabView | National Instruments | https://www.ni.com/en/shop/labview.html |

**1.3 Equipment**

| **equipment** | company | Catalogue number |
| --- | --- | --- |
| thermocouple | Pico Technologies | SE001 |
| Abbe ‘60’ refractometer | Abbe technologies | / |
| LED light source | Thorlabs | LED4D245 |
| LED driver | Thorlabs | DC4104 |
| 3D printer | Ultimaker | UMS3 |
| CNC mill | XYZ Machine tools | 500LR |
| Power meter | Thorlabs | PM100D |
| Power probe | Thorlabs | S130C |
| Microscope stage | Olympus | IX71 |
| 488 laser | Toptica | iBEAM-SMART-488 |
| 561 laser | Coherent | OBIS 561 |
| 640 laser | Cobolt | MLD 640 |
| Spatial light modulator | Forth Dimension Displays | SXGA-3DM |
| Pockels cell | Cornoptics | M350-80-01 |
| Camera | Hamamatsu | C11440 |

Supplementary Table S1: Summary of previous work on cold microscopy. Acronyms are : NA: Numerical Aperture, WD: Working Distance, WF: Widefield.

| **author** | **date** | **microscopy type** | **resolution** | **temperature reached** | **application** | **suitable for live biological application?** | **which stage if commercial** |
| --- | --- | --- | --- | --- | --- | --- | --- |
| **Li et al**[5] | **2020** | **Olympus WF microscope** | **~10 um from scale bar** | **-60°C to 25°C** | **water transport and intracellular ice in adipose tissue** | **yes** | **INSTEC-HCS302GXY,** |
| **Pach et al** [6] | **2017** | **optical microscope (WF), long WD objectives 10x, 20x, 50x** | **1-10um** | **0 -10°C** | **ice crystallisation dynamics** | **yes, for low-resolution work and for upright microscopes only** | **linkam LTS120E** |
| **Cheng et al** [10] | **2009** | **Confocal laser scanning microscopy (LSM 510) using a 63×/1.4-NA oil immersion objective lens or a 20× dry lens** | **1-10um from visual assessment of the scale bar** | **4°C with air objective; 8°C with oil objective** | **dendritic spines in neurons** | **yes** | **custom solution** |
| **Seki et al** [4] | **2008** | **optical microscope (WF) with a 20x objective, 500x magnification.** | **1-10um** | **-8 to -25°C** | **mouse oocyte intracellular ice** | **yes** | **Linkam BCS 196 cryostage** |
| **Buchner et al** [9] | **2007** | **Zeiss Axiovert 200M inverted microscope** | **~1um from visual assessment of the scale bar** | **−10°C to +95°C** | **plant cells - chloroplast protrusion** | **yes** | **custom solution** |

Supplementary Table S2: Parameters used for water, air and glass to compute the thermal models.

|  | Culture medium | Immersion Water | Air | Glass |
| --- | --- | --- | --- | --- |
| Density *ρ* (kg.m^-3^) | 999.89 | 998.19 | 1.293 | 2530 |
| Heat capacity C (J.kg^-1^.K^-1^) | 4184 | 4184 | 700 | 840 |
| Coefficient of heat conduction k (W.m^-1^.K^-1^) | 0.556 | 0.598 | 0.024 | 0.960 |
| Heat source Q (J.m^-2^.s^-1^) | 0 | 0 | 0 | 0 |
| Convective heat transfer h (W.m^-2^.K^-1^) | 650 | 650 | 55 | 0 |
| External temperature Text (°C) | 0 | 20 | 0 | 0 |

The model was run using the MATLAB PDE toolbox for 10,000 ms for contact with water and 30,000 ms for contact with air.

Supplementary Table S3:

Table of Student’s t-tests for the cross-temperature comparisons. The significant differences are highlighted in red.

|  | -2°C | 0°C | 2°C | 4°C | 6°C | 8°C | 10°C | 20°C | 30°C | 40°C |
| --- | --- | --- | --- | --- | --- | --- | --- | --- | --- | --- |
| -2°C | 1 | 0,249301 | 0,337341 | 0,471699 | 0,291509 | 0,465564 | 0,476021 | 0,253385 | 0,163356 | 0,030268 |
| 0°C |  | 1 | 0,145255 | 0,264706 | 0,11807 | 0,267216 | 0,278988 | 0,469662 | 0,369785 | 0,092893 |
| 2°C |  |  | 1 | 0,38549 | 0,452994 | 0,391803 | 0,318685 | 0,156504 | 0,089672 | 0,016638 |
| 4°C |  |  |  | 1 | 0,343437 | 0,494311 | 0,451069 | 0,259323 | 0,356349 | 0,381667 |
| 6°C |  |  |  |  | 1 | 0,348916 | 0,273314 | 0,126802 | 0,069113 | 0,011441 |
| 8°C |  |  |  |  |  | 1 | 0,444117 | 0,249934 | 0,179657 | 0,037725 |
| 10°C |  |  |  |  |  |  | 1 | 0,269311 | 0,182627 | 0,102362 |
| 20°C |  |  |  |  |  |  |  | 1 | 0,415602 | 0,112961 |
| 30°C |  |  |  |  |  |  |  |  | 1 | 0,135449 |
| 40°C |  |  |  |  |  |  |  |  |  | 1 |

Figure S1: parameters for thermal simulations


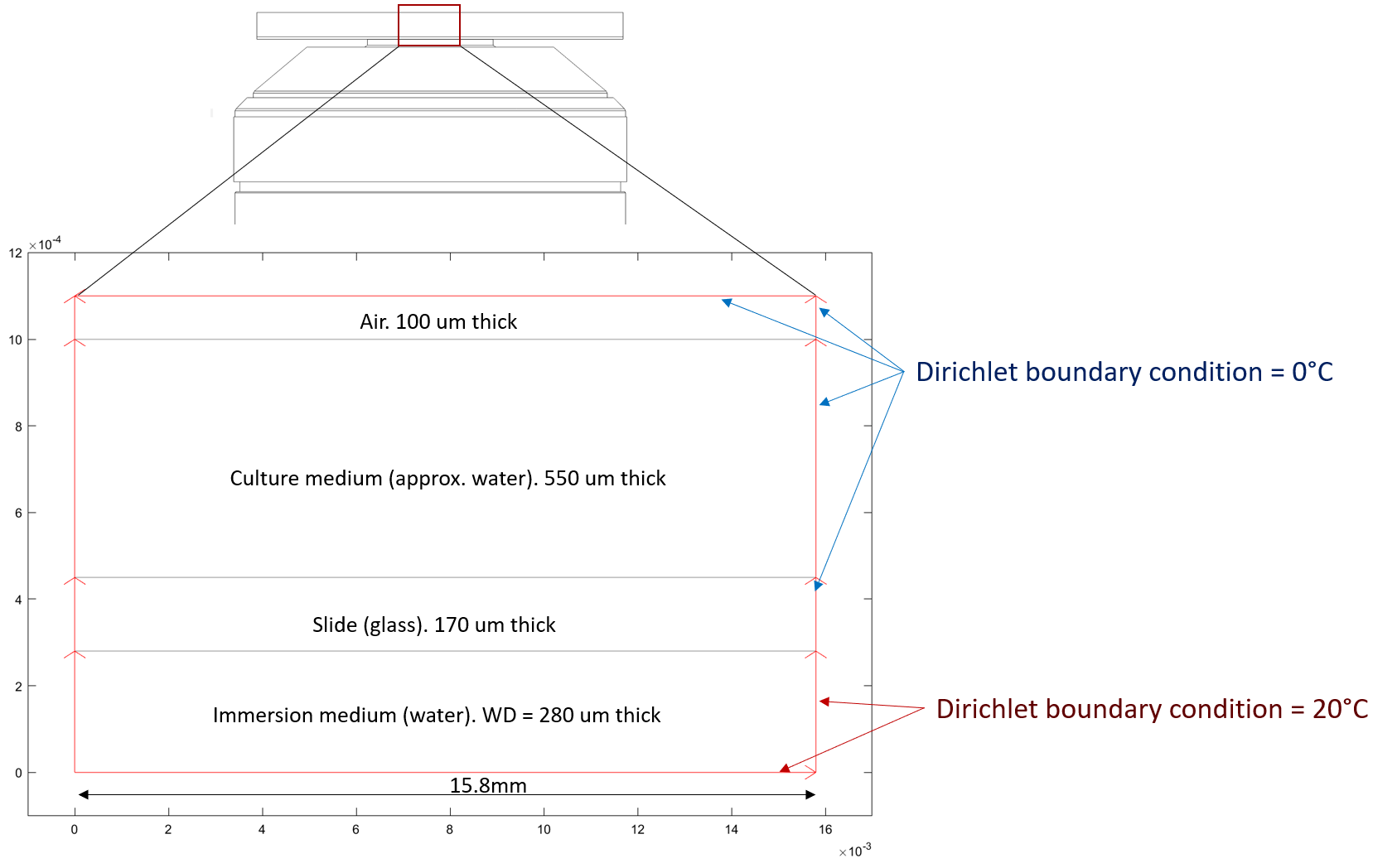


Figure S2: Renders of the cold incubator used to keep the out-of-focus sample cold. This is used in combination with the objective cooler.


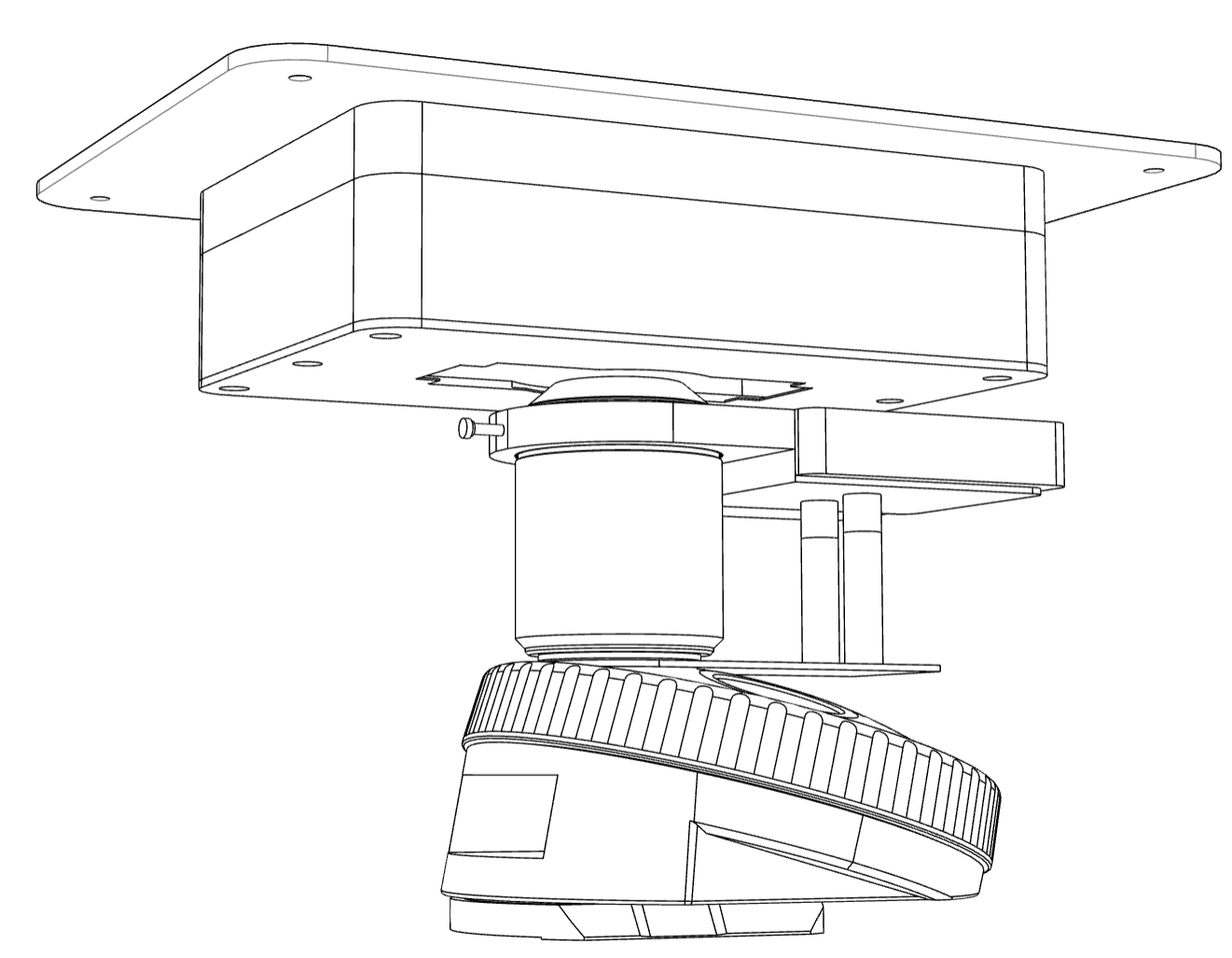

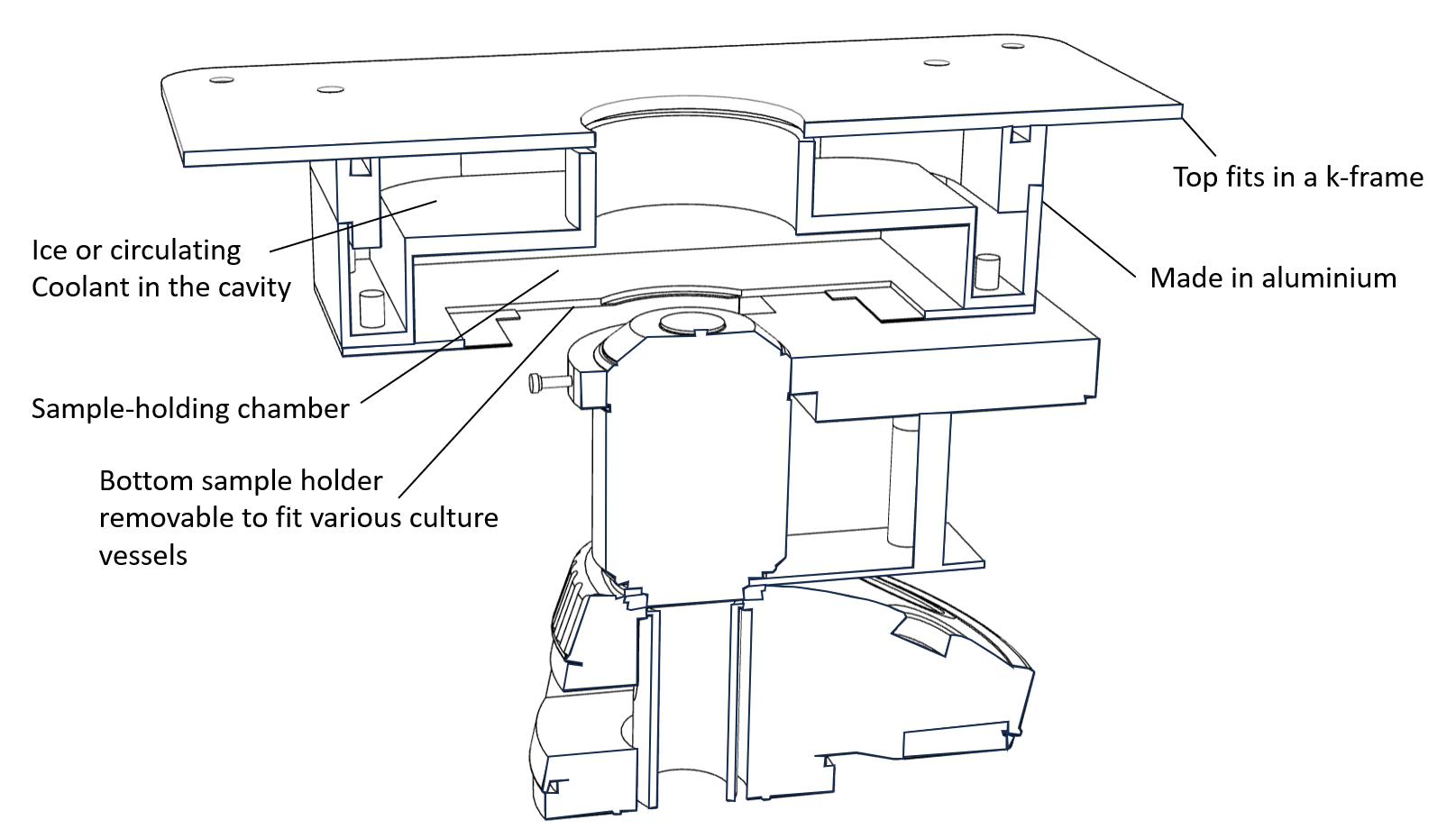


Figure S3: Fusion 360 thermal simulations to show that the collar needs to be cooled to -12.6°C for the focal volume to reach 0°C.


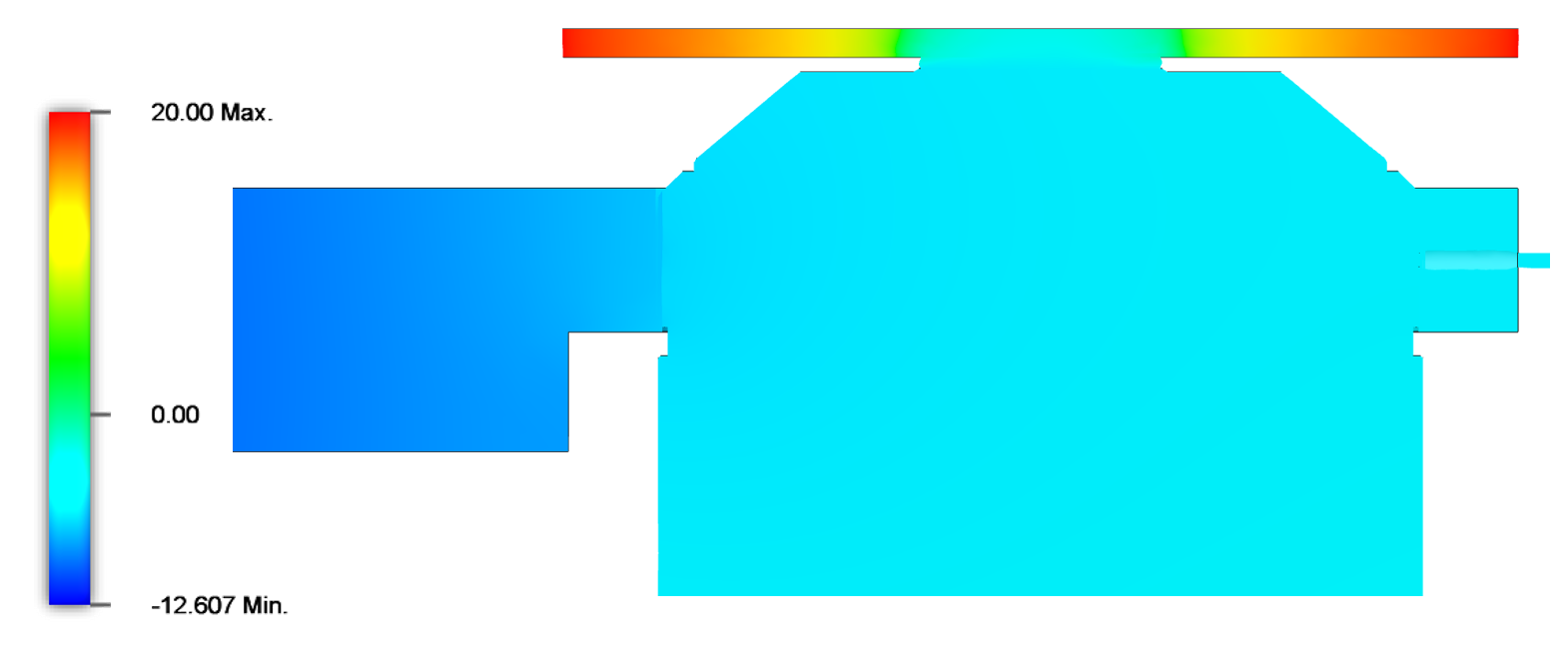


Figure S4: The weight of the collar introduces a noticeable tilt and needs to be offset by a support.


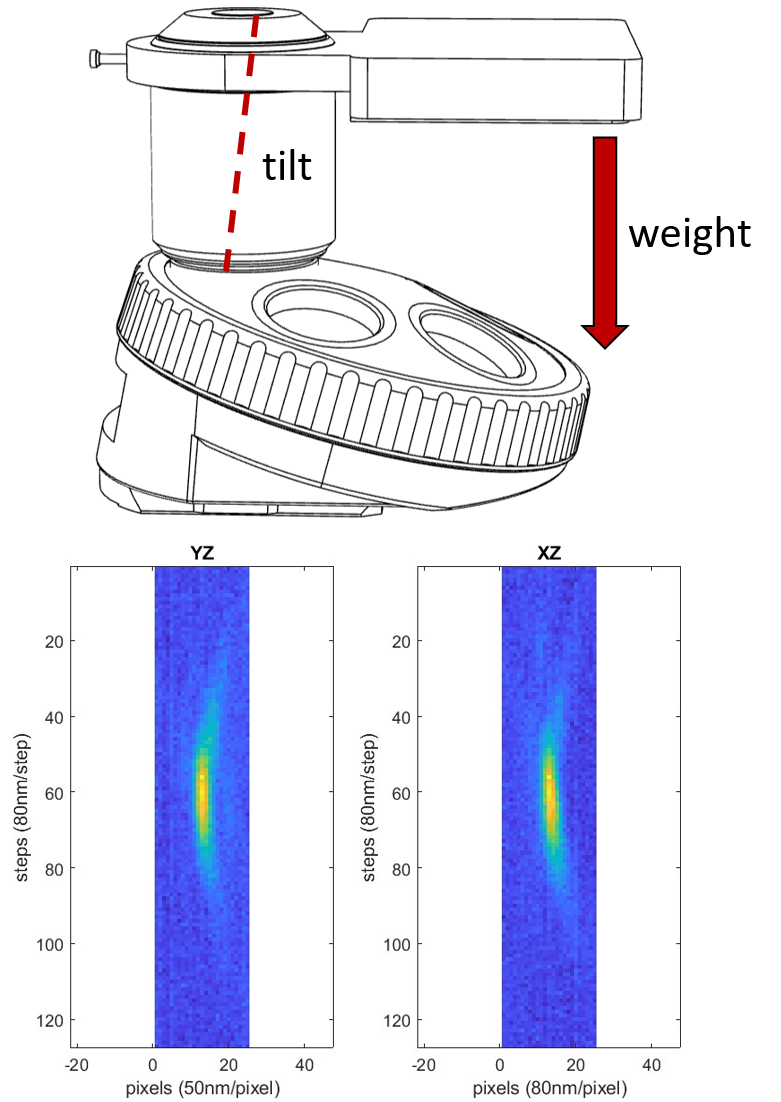

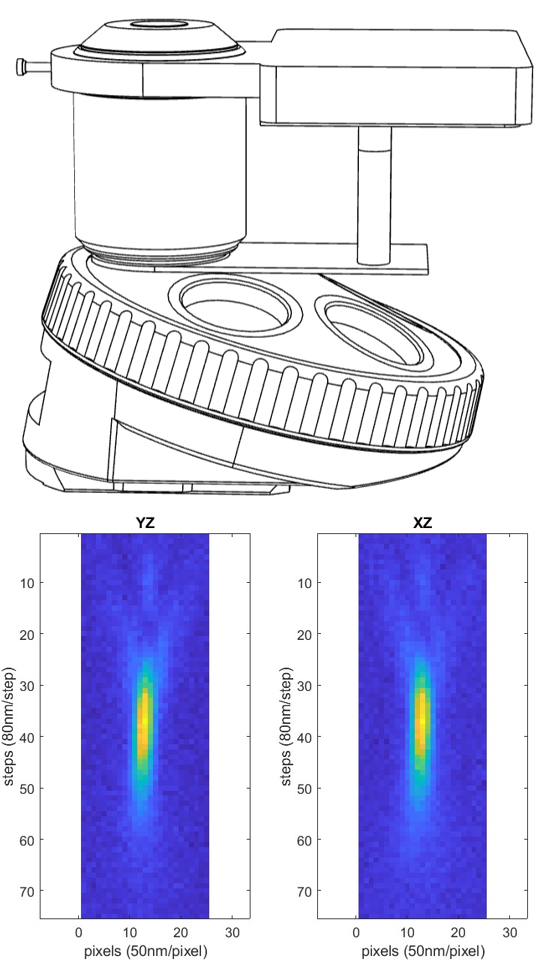


Figure S5: Candidates for sub-0°C immersion media.

1. 565 nm
2. 490nm
3. 625nm

Figure S6. Vibrations introduced by system modifications.

Amplitudes of vibrations introduced by our method remain within the resolution limit of the camera and mostly within one standard deviation of control conditions. a. Evolution of PSF line profiles over time for acquisition at high frame rate acquisition during active cooling of the collar. The amplitudes of vibrations are no greater than the camera resolution. b. Example fit to PSF line profile to determine the peak intensity position. c. Fast Fourier Transform (FFT) of the peak amplitude displacements from the origin (three repeats per condition). d. A frequency analysis of averages show that most of the recorded vibrations stay withing the standard deviation of the control and all remain below one pixel in magnitude.


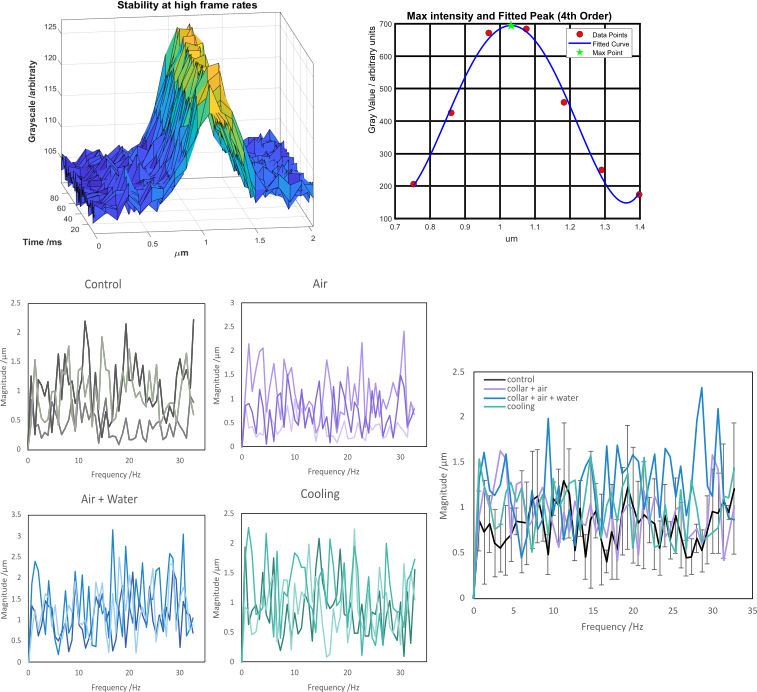


Figure S7. The performance stays stable over several months of use.

Fourier Ring Correlation analysis of images of fixed microtubules taken 6 months after another at 0°C with the same objective show no significant variation in performance. p= 0.46 (one-tailed unpaired Student’s t-test). Scale bars in both example images are 5µm.


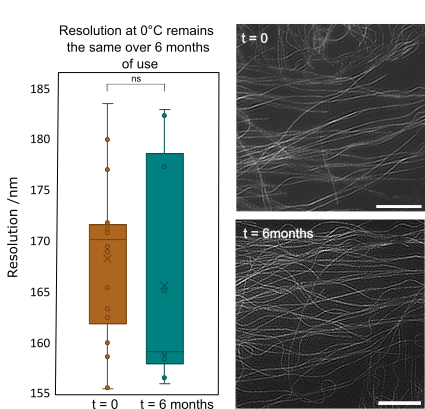

Supplement: Supplementary file 1 — Supporting Information [file SMTD-9-2401682-s001.docx]
